# Riboflavin: a scoping review for Nordic Nutrition Recommendations 2023

**DOI:** 10.29219/fnr.v67.10315

**Published:** 2023-12-22

**Authors:** Vegard Lysne, Hanna Sara Strandler

**Affiliations:** 1 Department of Health and Inequality, Norwegian Institute of Public Health, Oslo, Norway; 2 Swedish Food Agency, Uppsala, Sweden

**Keywords:** Riboflavin, Vitamin B2, dietary recommendations, flavoproteins

## Abstract

Only a few studies have explored relationships between riboflavin intake and function and a few studies have examined the effects of supplements on various clinical or biochemical outcomes. None of these studies, however, make a useful contribution to understanding requirements in healthy populations. Thus, there is no strong evidence to change the recommendations.

The requirement for riboflavin is estimated based on the relationship between intake and biochemical indices of riboflavin status, including urinary excretion and enzyme activities.

## Popular scientific summary

- Riboflavin (also known as vitamin B2) is essential for various biological functions, such as metabolism of several nutrients and is found mostly in animal-derived foods such as meat, cheese, eggs, and dairy.
- Riboflavin serves as a precursor for coenzymes needed for oxidative enzyme systems and electron transport pathways.
- Urinary riboflavin excretion is used as a marker of riboflavin status.
- Overt deficiency is rare, but susceptible groups include people with diets limiting animal-based foods, older people, people with high activity levels, and chronic alcohol abusers.
- Low intake may be related to health risks, including breast cancer and high blood pressure.

Riboflavin, formerly known as vitamin B2 due to being the second designated B-complex vitamin, is a yellow, fluorescent compound. Riboflavin functions as a precursor for the coenzymes riboflavin-5’-phosphate also called flavin mononucleotide (FMN), riboflavin-5’-adenosyl diphosphate also called flavin adenine dinucleotide (FAD), and as covalently bound flavin. These are essential components of several oxidative enzyme systems and participate in electron transport pathways (1). Riboflavin is also involved in the metabolism of several other nutrients such as niacin, vitamin B6, folate, cobalamin, vitamin D, and choline (2). Due to its involvement in a myriad of biological processes, by ensuring the catalytic activity and stability of flavoproteins, meeting riboflavin requirements is necessary to maintain human health and function. The main criteria for estimating riboflavin requirements are urinary riboflavin excretion, which is proportional to intake when tissues are saturated, and the activity coefficient of erythrocyte glutathione reductase (EGRAC) indicating the intracellular cofactor saturation.

Riboflavin is produced by bacteria, yeast, and some plants, but humans must obtain riboflavin through diet. The most abundant sources are animal-derived foods such as offal meats, cheese, eggs, and dairy. Riboflavin intake and status have been implicated concerning some health outcomes. Comparing high to low intakes has yielded an inverse association between riboflavin intake and breast cancer risk. Low-dose supplements, within the recommended intakes, have consistently been shown to lower blood pressure in individuals with a particular genetic variant and pharmacological doses have been suggested to prevent migraine headaches.

Although overt deficiency is rare, those on diets limiting or excluding animal-based foods may, therefore, be at an increased risk of not meeting the requirements. Other susceptible groups include the elderly, people with a high physical activity level, and chronic alcohol abusers. Suboptimal intakes have been frequently reported, also in high-income countries with abundant access to riboflavin-rich foods.

This scoping review aims to describe the totality of evidence for the role of riboflavin for health-related outcomes as a basis for setting and updating dietary reference values (DRVs) (Box 1).

*Box 1.* Background articles for Nordic Nutrition Recommendations 2023

- This article is one of many scoping reviews commissioned as part of the Nordic Nutrition Recommendations 2023 (NNR2023) project (3).
- The articles are included in the extended NNR2023 report but, for transparency, these scoping reviews are also published in Food and Nutrition Research.
- The scoping reviews have been peer reviewed by independent experts in the research field according to the standard procedures of the journal.
- The scoping reviews have also been subjected to public consultations (see report to be published by the NNR2023 project).
- The NNR2023 committee has served as the editorial board.
- While these articles are a main fundament, the NNR2023 committee has the sole responsibility for setting dietary reference values in the NNR2023 project.

## Methods

This review follows the protocol developed within the NNR2023 (3). The sources of evidence used in the scoping review follow the eligibility criteria described in the article ‘The Nordic Nutrition Recommendations 2022 – Principles and methodologies’(4). No *de novo* NNR2023 systematic reviews relevant for this scoping review were conducted (5). The main literature search for this scoping review was performed on June 11, 2021 in MEDLINE with a search string: (riboflavin[MeSH Terms] OR riboflavin[Title]) AND review(Publication Type) AND (‘2011’[Date - Publication] : ‘3000’[Date - Publication]) AND humans(Filter). The number of hits was 202. Based on the title, 10 articles were picked up, of which three were considered relevant based on the full articles (6–8). In addition, official reports published by EFSA (1, 9) and the Institute of Medicine (IOM) (10) were used, of which one was considered by the committee as a qualified systematic review (9). No strong evidence was identified in scientific literature since 2012 that likely would cause a change in DRVs. Neither was any topic related to a substantial health concern in the Nordic or Baltic countries identified.

## Physiology

**Fig. 1:**
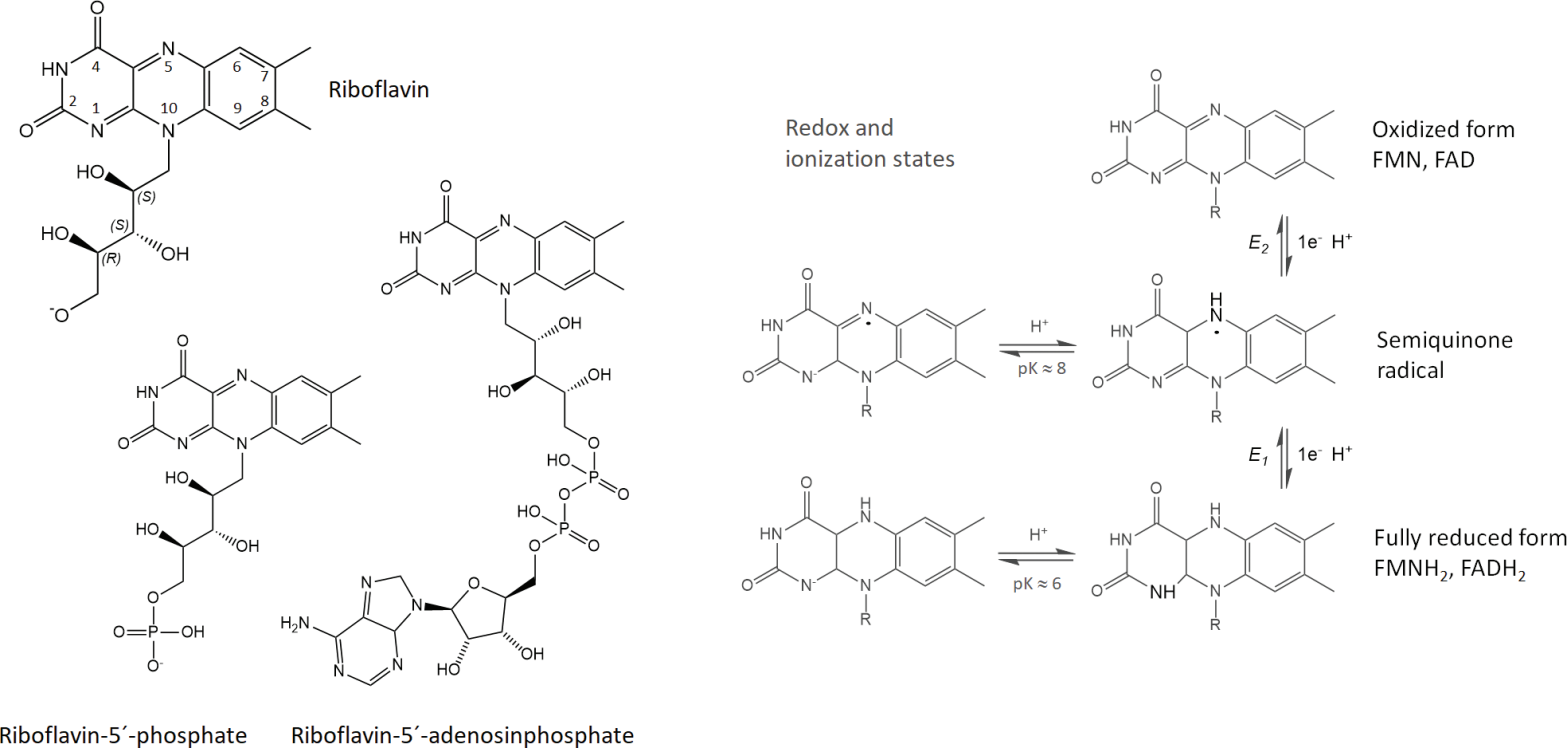
Riboflavin, riboflavin-5’-phosphate (FMN), and riboflavin-5’-adenosylphosphate (FAD). Redox and ionization states of the dimethyl-isoalloxazine system, R- ribityl- 5-phosphate (FMN) or ribityl-5’-adenosylphosphate (FAD). E1 is the midpoint redox potential for reduction of FMN semiquinone to hydroquinone (14). E2 is the midpoint redox potential for reduction of oxidized FMN to the semiquinone.

Riboflavin is a water-soluble vitamin with the molecular formula of C_17_H_20_N_4_O_6_ molecular mass of 376.4 g/mol and with a structure of substituted isoalloxazine with a sidechain of ribitol (Fig 1). The chemical name according to IUPAC is 7,8-dimethyl-10-[(2S,3S,4R)-2,3,4,5-tetrahydroxypentyl]benzo[g]pteridine-2,4-dione (11). Riboflavin, despite being a water-soluble vitamin, is sparingly soluble in water (10–13 mg/100 mL). It is also a very light-sensitive vitamin, degrading even in visible light, increasingly with pH and temperature. In the absence of light, the vitamin has optimum stability at pH 3.5–4 in aqueous solutions (12).

The main forms in the body are riboflavin-5´-phosphate (FMN) and riboflavin-5´-adenosyl diphosphate (FAD), (Fig 1). FMN and FAD, the abbreviations of the previous names, flavin mononucleotide and flavin adenine dinucleotide, are commonly accepted and used, although these compounds are not nucleotides. The term flavins is a generic name for all three forms of the vitamin. The term vitamin B2 can refer either to the substance riboflavin or to all flavins as a generic name (13).

**Fig. 2:**
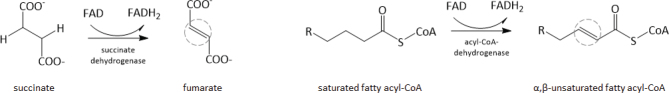
Examples of oxidation reactions with FAD as a coenzyme, succinate to fumarate and saturated fatty in the tricarboxylic acid cycle and of acyl-CoA to α,β‑unsaturated fatty acyl-CoA in fatty acid β-oxidation.

Flavins function as coenzymes; flavoproteins are enzymes with FMN or FAD as a cofactor. As coenzymes, FMN and FAD act as oxidizing agents. The conjugated ring structure of isoalloxazine allows different stable oxidation states, enabling the moiety to either donate or accept an electron (13, 15). FMN and FAD are the oxidized forms, FMNH_2_ and FADH_2_ are the reduced forms and the intermittent form is a semiquinone radical (Fig. 1) (14, 16). A transfer is possible of either one or two electrons, hydrogen atoms, or hydronium ions, and reactions forming a double bond involving FAD as a coenzyme (Fig 2). Most common is a non-covalent binding to the apo-protein. In rare cases riboflavin acts covalently bound as a prosthetic group, more commonly FAD is the coenzyme (16). A covalent binding enhances the redox potential (13, 15).

The majority of flavoproteins, 84%, use FAD as a cofactor; nitric oxide synthase is one of five that require both FMN and FAD as coenzymes (17). The majority of reactions are redox reactions; around ten percent of flavoproteins facilitate other types of reactions such as transferase, lyase, isomerase, and ligase reactions. Flavoproteins are involved in several metabolic pathways including the tricarboxylic acid cycle, fatty acid β‑oxidation (Fig. 2), amino acid catabolism, and the electron transport chain. Flavins also play a role in protein folding, DNA repair, and gene expression, and are required in chromatin remodeling, cell signaling as well as in the metabolism of other vitamins (13, 15).

In the de novo synthesis of niacin from tryptophan, kynurenine is converted to 3-hydroxykynurenine by the FAD-dependent kynurenine monooxygenase. In the metabolism of vitamin B6, the formation of pyridoxine phosphate requires an FMN-dependent oxidase (pyridoxine phosphate oxidase, PPOAC). In the folate metabolism FAD is a coenzyme for methylenetetrahydrofolate reductase (MTHFR), which influences the metabolism of homocysteine (13, 15). Low riboflavin status, assessed by EGRAC, has been associated with increased plasma homocysteine concentrations in subjects with the MTHFR C677T polymorphism, not seen in those with adequate riboflavin status (18). The intracellular metabolism of vitamin B12 depends on FMN or FAD as a cofactor for CblC, which is responsible for the initial intracellular release of free cobalamin for the generation of the active cobalamin cofactor forms (19). Flavins are also involved in the metabolism of vitamin D, cholesterol, and other steroids (20). In the choline oxidation pathway, FAD is needed as a cofactor for dimethylglycine and sarcosine dehydrogenase, involved in the oxidation of choline ultimately yielding glycine.

**Fig. 3:**
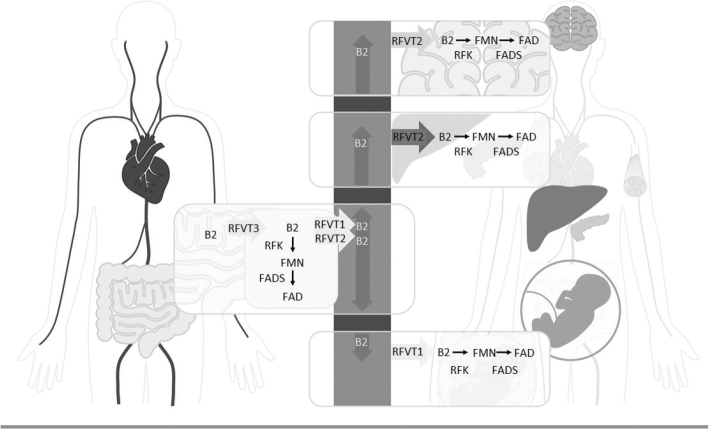
Riboflavin metabolism, modified from Mosegaard et al. (2). Riboflavin is absorbed from the gastrointestinal tract predominantly by riboflavin transporter 3 (RFVT3). Inside the gastrointestinal cells, riboflavin can either be further metabolized to flavin mononucleotide (FMN) by riboflavin kinase (RFK) or to flavine adenine dinucleotide (FAD) by FAD synthase (FADS) or transported to the bloodstream by riboflavin transporter 1 (RFVT1) and riboflavin transporter 2 (RFVT2). Riboflavin is distributed via the bloodstream to its destination cells. In addition to being expressed in the gastrointestinal system, RFVT1 is expressed in the placenta, where it carries riboflavin from the maternal to fetal bloodstream. RFVT2 is expressed not only all over the body and highly expressed in the brain, endocrine organs, such as the pancreas but also in the liver and muscle tissue. Inside the destination cells, riboflavin is used directly or transformed into either FMN or FAD, which function as cofactors in several processes. Several factors can affect human riboflavin status, hereunder, genetics, inflammation and infections, exercise, diet and nutrition, aging, and pregnancy. The figure is adapted from Mosegaard et al. (2).

Our main source of flavins is diet. In the large intestine, bacteria produce flavins utilized by colonic epithelial cells. However, it is unknown if microbial flavins are available as a source for riboflavin metabolism (21). When ingested, flavins are released from proteins by gastric acid and free riboflavin is released from phosphate bound sidechains by ileac brush border enzymes, alkaline phosphatase, FMN-phosphatase, and FAD-diphosphate. Free riboflavin is then absorbed via a specific transport mechanism in the small intestine [22, 23, 24]. This mechanism is saturated at doses of about 30–50 mg. Absorption rates of free riboflavin are reported to be 50–60% at doses of 2–25 mg, and absorption rates of riboflavin from whole foods are 60–70% (13, 14). Specific transporters facilitate uptake by plasma and mitochondrial membrane, and riboflavin is converted to FAD or FMN by enzymes (Fig. 3) (2, 24). Conversion of riboflavin to FMN and FAD is regulated by thyroid hormone (13).

Riboflavin reserves are mainly in flavoproteins and to a lesser degree as free riboflavin. Changes in nitrogen balance can therefore affect urinary excretion. The urinary excretion of riboflavin can increase under conditions of negative nitrogen balance or during infections, but the opposite can be seen during periods of rapid growth (25). No consistent relationship has been found between riboflavin requirement, measured by urine excretion or retention, and protein intake in situations of positive protein balance. Reabsorption in the kidney contributes to riboflavin homeostasis (13).

Conditions that increase metabolism and energy demand, such as exercise or periods of growth, activate reactions and enzymes involving FAD and FMN and hence increase the need for riboflavin. The need may also increase with age due to impairment in absorption, and insufficient intakes have been reported in the elderly population (2). Inflammation and infections activate the immune system and consequently increase energy production for cellular immune responses and the production of reactive oxygen species. The vitamin also takes part in the activation of macrophages and has anti-inflammatory effects. Besides an increased energy demand, normal development of the fetus and baby requires sufficient maternal vitamin status during pregnancy and breastfeeding (2, 15). During pregnancy sufficient riboflavin to meet the metabolic needs of the fetus is provided by an estrogen-dependent riboflavin carrier protein (13).

Several inborn errors of metabolism respond well to riboflavin therapy, such as multiple acyl-CoA dehydrogenation deficiency (MADD), and disorders of riboflavin transporters (2, 26). Small-case studies and case reports have shown promising results for other inborn errors, warranting further investigation.

## Assessment of nutrient status

Static markers of riboflavin status include urinary riboflavin, and plasma or erythrocyte riboflavin, FAD, and FMN. Functional markers include the activity coefficient for EGRAC or PPOAC measured *in vitro* using red blood cells (1).

As the body has a limited ability to store riboflavin, intakes above tissue reserve capacity are excreted in the urine, and the urinary excretion is proportional to the intake (1). Depletion-repletion studies show that the urinary excretion of riboflavin increases gradually with increasing intakes, and tissue saturation is indicated by a sharp increase at intakes above about 1 mg/day. EFSA (1) considers 24 h or fasting urinary excretion of riboflavin, with or without correction for creatinine excretion, as a suitable marker of short-term riboflavin intake and status. However, for intakes <1.1 mg/day, urinary riboflavin excretion is not a sensitive marker. Data from depletion-repletion studies have also been used to propose cut-off values for deficiency (<40 µg/day, <27 µg/g creatinine), insufficiency (40–120 µg/day or 80 µg/g creatinine), and adequacy (>120 µg/day) for adults (1).

The EGRAC is the ratio of enzyme activity measured *in vitro*, with and without the addition of exogenous FAD. A ratio of 1.0 indicates an absence of stimulation, indicating complete cofactor saturation by intracellular FAD, while values above 1 indicate incomplete endogenous saturation. EGRAC below 1.3 are considered to indicate adequate status, based on the relationship between EGRAC and urinary excretion (1). One limitation of EGRAC is that it cannot be used in individuals with glucose-6-phosphate dehydrogenase (G6PD)-deficiency, as their EGR have an increased avidity for FAD, which prevents the detection of low riboflavin status.

No cut-off values have been suggested for plasma concentrations of riboflavin, FAD, and FMN, for the assessment of riboflavin deficiency or adequacy (1). The main reason is that these markers are highly sensitive toward recent dietary intake, and they are also not correlated with EGRAC, thus limiting their usefulness for the assessment of riboflavin deficiency and adequacy. Erythrocyte riboflavin, FAD, and FMN have been suggested to reflect longer-term riboflavin intake, and they are correlated with EGRAC. However, albeit several cut-off values have been suggested, EFSA concluded that the underlying data were insufficient to make any firm conclusions (1). Like EGRAC, the PPOAC is the ratio of enzyme activity measured *in vitro*, with and without the addition of exogenous FMN. PPOAC has been considered as a promising alternative to EGRAC in individuals with, or in populations with a high prevalence of, G6PD-deficiency. However, criteria for the assessment of riboflavin status with PPOAC have not yet been developed (1).

## Dietary intake in Nordic and Baltic countries

Major sources of riboflavin in the Nordic diets are milk and dairy products, meat, and meat products. The average dietary intake according to national dietary surveys is in the range of 1.8 mg–2.3 mg/10 MJ in the Nordic countries and 1.4–1.7 mg/10 MJ in the Baltic countries (see scoping review on dietary intake in Nordic countries). The main dietary sources of riboflavin are animal-derived foods, suggesting that riboflavin sufficiency may be a concern among those excluding or limiting these foods, i.e. vegetarians and vegans. Non-animal sources include legumes, nuts, green vegetables, and fortified plant-based milk alternatives, whilst grain products are relatively poor sources unless they are enriched or fortified. It has been reported that vegan diets, in particular, may not be able to supply sufficient amounts of riboflavin (27), although intake has also been reported to be similar between omnivores, vegetarians, and vegans (28). These divergences may reflect differences in diets in the different study populations and also suggest that it is possible to adequately meet the requirement of riboflavin with vegan diets. Furthermore, riboflavin has been listed as a potential nutrient of concern for infants on macrobiotic diets, which are mostly plant-based (29).

## Health outcomes relevant for Nordic and Baltic countries

### Deficiency

The clinical signs of riboflavin deficiency are unspecific and include sore throat, hyperemia and edema of the pharyngeal and oral mucous membranes, various skin changes including angular stomatitis, seborrheic dermatitis, and glossitis, and normocytic anemia (1, 10). Isolated dietary riboflavin deficiency usually does not occur, and deficiency is normally seen in association with other nutritional deficiencies. However, riboflavin intakes considered insufficient are often reported and poor riboflavin status representing subclinical but not overt deficiency has been observed in children and adolescents in high-income countries with abundant access to riboflavin rich foods (2). Those excluding animal-derived foods may be particularly susceptible to suboptimal intakes. Furthermore, elderly people with limited diets are reported to have suboptimal B-vitamin status, and supplementing otherwise healthy subjects aged > 50 years with 5 mg/day riboflavin resulted in improved riboflavin status as indicated by a reduced EGRAC (30). Due to metabolic stress, even short periods of increased physical activity may negatively impact riboflavin status. Thus, those with a high physical activity level may be at increased risk of developing riboflavin deficiency (2). A high prevalence of riboflavin deficiency has been reported in hemodialysis patients, and chronic alcohol abusers, suggested to be due to alcohol interfering with the release of riboflavin from dietary FAD and FMN (1). Individuals with inborn errors of metabolism affecting riboflavin metabolism may be at particular risk due to increased riboflavin requirements (2).

### Toxicity

There are no reports of adverse effects of high riboflavin intakes from dietary sources or supplements (1). The limited studies in which large doses (100–400 mg/day) of supplemental riboflavin have been administered do not indicate any adverse effects (31). However, these studies only included self-reported assessment of adverse effects and no objective biochemical markers. Hence, there are insufficient data to set a UL for riboflavin.

### Hypertension

Genome-wide association studies, as well as epidemiological studies, have linked the gene encoding for methylenetetrahydrofolate reductase (MTHFR) to hypertension. The MTHFR C667T polymorphism has been consistently associated with hypertension, and low-dose supplementation of riboflavin (1.6 mg/day) has been reported to modify the association (8). Whether there is an added benefit of higher doses is not currently known. Three clinical trials of riboflavin supplementation have demonstrated a substantial blood pressure reduction of 6–13 mmHg among patients with the MTHFR 677TT (homozygous) genotype when receiving riboflavin supplements for 16 weeks (32–34).

### Breast cancer

In a systematic review and meta-analysis of 10 epidemiological studies (five case-control and five prospective cohorts), comprising a total of 12,268 breast cancer cases, Yu et al. reported a relative risk (95% CI) of 0.85 (0.76, 0.95) when comparing the highest (mean 2.5 mg/day) to the lowest (mean 1.1 mg/day) dietary riboflavin intakes (7). This association was robust to sensitivity analyses based on geographical location, study design, and statistical adjustment for family history of breast cancer. The reported dose-response relative risk estimate was 0.94 (0.90, 0.99) per 1 mg/day increment in riboflavin intake.

### Migraine

In a systematic review of 11 clinical trials, Thompson et al. reported that supplemental riboflavin in high doses ranging from 50 to 400 mg/day prevented migraine headaches in some adults, while results in children and adolescents remained inconclusive (6). The supplement was in general well tolerated.

## Requirement and recommended intakes

The requirement for riboflavin is estimated based on the relationship between intake and biochemical indices of riboflavin status, including urinary excretion and EGRAC. Generally, riboflavin metabolism and intake are related to energy intake at normal intake ranges in populations such as those of the Nordic countries. However, at low energy intakes (below 8 MJ/day) the requirement expressed per MJ might be higher and the opposite might be the case at energy intakes well above 12 MJ/day. Urinary excretion is generally decreased and EGRAC increased when physical activity is increased, indicating that requirements are modified by the level of physical activity and energy expenditure. Furthermore, requirements may be higher in individuals carrying the MTHFR 677TT genotype.

In NNR 2012 the average requirement (AR) was estimated to be 0.12 mg/MJ, based on older studies using urinary excretion and EGRAC as the main criteria (35). The recommended intake (RI) was set to 0.14 mg/MJ, corresponding to RI’s of 1.5–1.6 and 1.2–1.3 mg/day for adult men and women, respectively. It was stressed that when planning diets, riboflavin intake should not be below 1.2 mg/day even at low energy intakes (below 8MJ/day), and the LI was set to 0.8 mg/day based on depletion/repleting studies. For pregnant and lactating women, an extra 0.3 and 0.4 mg/day, respectively, was recommended.

The U.S. DRI’s were based on a combination of urinary excretion, circulating riboflavin, and EGRAC, and are expressed in absolute intakes (10). They reported RDA’s of 1.3 and 1.1 mg/day for adult men and women, respectively. The RDA for children and adolescents was extrapolated from adult values. It was emphasized that those undergoing dialysis treatment or having severe malabsorption, were likely to require more riboflavin.

EFSA considers the inflection point of the urinary excretion curve as the most sensitive criteria for riboflavin requirement, occurring for intakes between 1.1 and 1.6 mg/day, with EGRAC as a supportive biomarker interpreting values of 1.3 or less as indicating sufficiency (1). The same AR of 1.3 mg/day, and a population RI of 1.6 mg/day, was set for men and women, as they concluded that there was no indication of different requirements across sex. The reported population RI was assumed to cover the variation in requirement due to physical activity and MTHFR genotype.

## References

[1] Turck D, Bresson J, Burlingame B, Dean T, Fairweather–Tait S, Heinonen M, et al. Dietary reference values for riboflavin. EFSA J. 2017; 15(8): 4919. doi: 10.2903/j.efsa.2017.4919 PMC7010026 32625611

[2] Mosegaard S, Dipace G, Bross P, Carlsen J, Gregersen N, Olsen RKJ. Riboflavin deficiency – implications for general human health and inborn errors of metabolism. Int J Mol Sci. 2020; 21(11); 3847. doi: 10.3390/IJMS21113847 32481712 PMC7312377

[3] Blomhoff R, Andersen R, Arnesen EK, Christensen JJ, Eneroth H, Erkkola M, et al. Nordic nutrition recommendations 2023. Nordic Council of Ministers; 2023. Available from: https://pub.norden.org/nord2023-003 [cited 16 October 2023].

[4] Christensen JJ, Arnesen EK, Andersen R, Eneroth H, Erkkola M, Høyer A, et al. The Nordic nutrition recommendations 2022 – principles and methodologies. Food Nutr Res. 2020; 64. doi: 10.29219/fnr.v64.4402 PMC7307430 32612489

[5] Høyer A, Christensen JJ, Arnesen EK, Andersen R, Eneroth H, Erkkola M, et al. The Nordic nutrition recommendations 2022 – prioritisation of topics for de novo systematic reviews. Food Nutr Res. 2021; 65. doi: 10.29219/FNR.V65.7828 PMC8897982 35291553

[6] Thompson DF, Saluja HS. Prophylaxis of migraine headaches with riboflavin: a systematic review. J Clin Pharm Ther. 2017; 42(4): 394–403. doi: 10.1111/jcpt.12548 28485121

[7] Yu L, Tan Y, Zhu L. Dietary vitamin B2 intake and breast cancer risk: a systematic review and meta-analysis. Arch Gynecol Obs. 2017; 295(3): 721–9. doi: 10.1007/s00404-016-4278-4 28035488

[8] McNulty H, Strain JJ, Hughes CF, Ward M. Riboflavin, MTHFR genotype and blood pressure: a personalized approach to prevention and treatment of hypertension. Mol Aspects Med. 2017; 53: 2–9. doi: 10.1016/j.mam.2016.10.002 27720779

[9] Buijssen M, Eeuwijk J, Vonk Noordegraaf-Schouten M. Literature search and review related to specific preparatory work in the establishment of dietary reference values for Riboflavin. EFSA Support Publ. 2014; 11(5): 591E. doi: 10.2903/SP.EFSA.2014.EN-591

[10] Institute of Medicine. Dietary reference intakes: The essential guide to nutrient requirements. Washington, DC: National Academies Press; 2006.

[11] De Gruyter. Riboflavin. 2016. Available from: https://www.degruyter.com/document/database/IUPAC/entry/iupac.compound.493570/html [cited 2021 Jun 21].

[12] Ball GFM. Vitamins in foods: Analysis, bioavailability, and stability. 1st ed. 2006. CRC Press. doi: 10.1201/9781420026979

[13] Ball GFM. Flavins: Riboflavin, FMN and FAD (Vitamin B2). In: Vitamins; Their role in the human body. Oxford, UK: Blackwell Publishing Ltd; 2004, p. 289–300. doi: 10.1002/9780470774571.ch12

[14] Mayhew SG. The effects of pH and semiquinone formation on the oxidation-reduction potentials of flavin mononucleotide. A reappraisal. Eur J Biochem. 1999; 265(2): 698–702. doi: 10.1046/J.1432-1327.1999.00767.X 10504402

[15] Zempleni J, Suttie JW, Gregory JF, Patrick I, Stover J. Handbook of vitamins, Boca Raton, Florida, USA: CRC Press, 2013. doi: 10.1201/b15413

[16] Rich PR, Maréchal A. Electron transfer chains: Structures, mechanisms and energy coupling. In: Egelman EH, ed. Comprehensive biophysics, Oxford: Elsevier, 2012, p. 72–93.

[17] Lienhart WD, Gudipati V, MacHeroux P. The human flavoproteome. Arch Biochem Biophys. 2013; 535(2): 150–62. doi: 10.1016/J.ABB.2013.02.015 23500531 PMC3684772

[18] McNulty H, McKinley MC, Wilson B, McPartlin J, Strain JJ, Weir DG, et al. Impaired functioning of thermolabile methylenetetrahydrofolate reductase is dependent on riboflavin status: implications for riboflavin requirements. Am J Clin Nutr. 2002; 76(2): 436–41. doi: 10.1093/ajcn/76.2.436 12145019

[19] Koutmos M, Gherasim C, Smith JL, Banerjee R. Structural basis of multifunctionality in a vitamin B12-processing enzyme. J Biol Chem. 2011; 286(34): 29780–7. doi: 10.1074/JBC.M111.261370 21697092 PMC3191019

[20] Pinto JT, Cooper AJL. From cholesterogenesis to steroidogenesis: role of riboflavin and flavoenzymes in the biosynthesis of vitamin D. Adv Nutr. 2014; 5(2): 144–63. doi: 10.3945/AN.113.005181 24618756 PMC3951797

[21] Said HM. Recent advances in transport of water-soluble vitamins in organs of the digestive system: a focus on the colon and the pancreas. Am J Physiol Gastrointest Liver Physiol. 2013; 305(9): G601–10. doi: 10.1152/AJPGI.00231.2013 23989008 PMC3840235

[22] Rivlin RS. Riboflavin metabolism. N Engl J Med. 1970; 283(9): 463–72. doi: 10.1056/nejm197008272830906 4915004

[23] Said HM. Intestinal absorption of water-soluble vitamins in health and disease Biochem J. 2011; 437(3): 357–72. doi: 10.1042/BJ20110326 21749321 PMC4049159

[24] Barile M, Giancaspero TA, Leone P, Galluccio M, Indiveri C. Riboflavin transport and metabolism in humans. J Inherit Metab Dis. 2016; 39(4): 545–57. doi: 10.1007/S10545-016-9950-0 27271694

[25] Sauberlich HE. Vitamin metabolism and requirements: some aspects reviewed. S Afr Med J. 1975; 49(54): 2235–44. 1209445

[26] Balasubramaniam S, Christodoulou J, Rahman S. Disorders of riboflavin metabolism. J Inherit Metab Dis. 2019; 42(4): 608–19. doi: 10.1002/JIMD.12058 30680745

[27] Bakaloudi DR, Halloran A, Rippin HL, Oikonomidou AC, Dardavesis TI, Williams J, et al. Intake and adequacy of the vegan diet. A systematic review of the evidence. Clin Nutr. 2021; 40(5): 3503–21. doi: 10.1016/J.CLNU.2020.11.035 33341313

[28] Neufingerl N, Eilander A. Nutrient intake and status in adults consuming plant-based diets compared to meat-eaters: a systematic review. Nutrients. 2021; 14(1): 29. doi: 10.3390/NU14010029 35010904 PMC8746448

[29] Kiely ME. Risks and benefits of vegan and vegetarian diets in children. Proc Nutr Soc. 2021; 80(2): 159–64. doi: 10.1017/S002966512100001X 33504371

[30] Heffernan M, Doherty LC, Hack Mendes R, Clarke M, Hodge S, Clements M, et al. Effectiveness of a fortified drink in improving B vitamin biomarkers in older adults: a controlled intervention trial. Nutr Metab (Lond). 2021; 18(1): 104. doi: 10.1186/S12986-021-00630-8 34876175 PMC8650259

[31] European Food Safety Authority. Tolerable upper intake levels for vitamins and minerals. 2006, Available from: http://www.efsa.europa.eu/en/ndatopics/docs/ndatolerableuil.pdf

[32] Horigan G, McNulty H, Ward M, Strain JJJ, Purvis J, Scott JM. Riboflavin lowers blood pressure in cardiovascular disease patients homozygous for the 677C→T polymorphism in MTHFR. J Hypertens. 2010; 28(3): 478–86. doi: 10.1097/HJH.0b013e328334c126 19952781

[33] Wilson CP, Ward M, McNulty H, Strain JJ, Trouton TG, Horigan G, et al. Riboflavin offers a targeted strategy for managing hypertension in patients with the MTHFR 677TT genotype: a 4-y follow-up. Am J Clin Nutr. 2012; 95(3): 766–72. doi: 10.3945/ajcn.111.026245 22277556

[34] Wilson CP, McNulty H, Ward M, Strain JJ, Trouton TG, Hoeft BA, et al. Blood pressure in treated hypertensive individuals with the mthfr 677tt genotype is responsive to intervention with riboflavin: findings of a targeted randomized trial. Hypertension. 2013; 61(6): 1302–8. doi: 10.1161/HYPERTENSIONAHA.111.01047 23608654

[35] Nordic Council of Ministers. Nordic Nutrition Recommendations 2012 – Integrating nutrition and physical activity. 5th ed. 2014, Available from: https://www.norden.org/en/publication/nordic-nutrition-recommendations-2012

